# Phenotype Classification of Zebrafish Embryos by Supervised Learning

**DOI:** 10.1371/journal.pone.0116989

**Published:** 2015-01-09

**Authors:** Nathalie Jeanray, Raphaël Marée, Benoist Pruvot, Olivier Stern, Pierre Geurts, Louis Wehenkel, Marc Muller

**Affiliations:** 1 GIGA-Development, Stem Cells and Regenerative Medicine, Organogenesis and Regeneration, University of Liège, Liège, Belgium; 2 GIGA-Systems Biology and Chemical Biology, Dept. EE & CS, University of Liège, Liège, Belgium; 3 GIGA Bioinformatics Core Facility, University of Liège, Liège, Belgium; National University of Singapore, SINGAPORE

## Abstract

Zebrafish is increasingly used to assess biological properties of chemical substances and thus is becoming a specific tool for toxicological and pharmacological studies. The effects of chemical substances on embryo survival and development are generally evaluated manually through microscopic observation by an expert and documented by several typical photographs. Here, we present a methodology to automatically classify brightfield images of wildtype zebrafish embryos according to their defects by using an image analysis approach based on supervised machine learning. We show that, compared to manual classification, automatic classification results in 90 to 100% agreement with consensus voting of biological experts in nine out of eleven considered defects in 3 days old zebrafish larvae. Automation of the analysis and classification of zebrafish embryo pictures reduces the workload and time required for the biological expert and increases the reproducibility and objectivity of this classification.

## Introduction

Zebrafish (*Danio rerio*) is commonly used as a vertebrate model organism [1] which has first shown its usefulness in the field of embryonic developmental biology, but also, during the last years, increasingly in toxicology, pharmacology and vertebrate behavioral biology [2, 3]. It is fitted just as well to physiological analyses than genetic ones. Studies performed during the last twenty years revealed a remarkable similarity in the genetic and metabolic pathways between fish and mammals, opening up the way to the generation of fish models for many human pathologies [4].

Thanks to the aquatic life mode, the permeability to small molecules, the low cost and the transparency of the zebrafish embryos, the effect of chemical substances on their development can be studied by simple microscopic observation. Several hundreds of embryos can easily be obtained and used to assess the effects of a large number of substances in a screening approach.

Lethality and teratogenicity tests represent by far the most used tests and will always be a prerequisite for further, more specific assays. In the European “REACH” directive {Council, 2006 #4255}, the “Zebrafish EmbryoToxicity” (ZET) test is included as an alternative to animal testing {Testing, 2014 #4254} and is bound for use on a large number of molecules. In view of the large number of tests that will have to be performed, we identified the observation phase as the main rate- and quality-limiting step in the procedure. Classically, an expert evaluates the defects caused by a chemical compound by directly observing each embryo under a microscope and scoring manually the different effects. Typical defects are illustrated by photography and the observations are statistically processed to infer the toxicological effects of the drug. Ideally, all the evaluated individuals should be documented and classified according to the defects observed. This method is tedious, time-consuming and prone to appreciation subjectivity which can affect the reproducibility of the results. According to [5], an expert is restricted to visualization of a mean number of 400 specimens a day. Knowing that a single female is able to produce up to 800 eggs per day, manual observation is clearly the limiting step. The large number of substances to be tested and the need for accuracy of the results call for methods allowing automation of data acquisition as well as identification of the defects and classification of the acquired images.

From a recent survey, it clearly appears that the aim of a fully automatic procedure to capture images of zebrafish larvae and classify/quantify their phenotypes is not yet reached [6]. Here, we propose an efficient and flexible approach for defect classification based on supervised learning algorithms using a limited learning set of images annotated by experts to perform efficient and reliable automatic classification of various defects caused by teratogenic chemicals in 3 days old zebrafish larvae. This tool carries the potential to be adapted to different types of images (different stages, fluorescent) in future applications.

## Materials and Methods

### Fish and embryo maintenance

Zebrafish (*Danio rerio*) of the AB strain were reared in a recirculating system from Techniplast, Italy at a maximal density of 7 fish/l. The water characteristics were as follows: pH = 7.4, conductivity = 500 µScm-1, temperature = 28°C. The light cycle was controlled (14 h light, 10 h dark). Standard references of breeding can be found in [7]. Fish were fed twice daily with dry powder (ZM fish food) adapted to their age and once daily with fresh *Artemia salina* nauplii (ZM fish food). Larvae aged less than 14 days were also fed twice daily with a live paramecia culture. Wild type embryos were used and staged according to Kimmel et *al* [8].

The day before breeding, wild-type adult male and female zebrafish were set up in several breeding tanks, separated by a clear plastic wall. After the light was turned on the next morning, walls are removed and eggs are generated by natural mating and then, collected from 30 minutes to 2 hours after spawning. After sorting, clean eggs are moved to Petri dishes and incubated at 28°C in E3 medium (5 mM Na Cl, 0.17 mM KCl, 0.33 mM CaCl_2_, 0.33 mM MgSO_4_, 0.00001% Methylene Blue).

Animal care and all experimentation were conducted in compliance with Belgian and European laws (Authorization: LA1610002). All experiments and the entire study were evaluated by the Ethical Committee of the University of Liège, Belgium and the study protocols were approved under the file numbers 1076 and 13–1506.

### Chemicals and treatments

All chemicals used for zebrafish embryo treatment were purchased from Sigma-Aldrich (Diegem, Belgium). Stock solutions were prepared according to the manufacturer’s instructions concerning solubility. Propanolol was dissolved in dimethyl sulfoxide (DMSO) to obtain a 1 M stock solution. Amiodarone was dissolved in ethanol (ETOH) to obtain a 1 M stock solution. Acetaminophen was dissolved in ETOH 100% to make a 500 mM stock solution. Valproic acid (VPA) was dissolved in pure water to make a 1 mM stock solution. The heavy metals: thallium, methylmercury (MeHg), lead acetate (PbAc) and zinc sulfate (ZnSO4) were dissolved in pure water to obtain respectively 10 g/l, 100 mg/l, 100 mg/l, 100 mg/l stock solutions. Caffeine, theophyllin and 4,5-dichloroanilin (DCA) were prepared as, respectively 82.4 mM, 40.85 mM and 200mg/l stocks in pure water.

The treatment solutions for toxicity tests were obtained by dilution of the stock solutions in embryo medium (E3). Zebrafish embryos were treated at 2 dpf (days post fertilization) in batches of 25 individuals distributed into 6-well plates and analyzed after 24 hours of treatment. At this stage (3 days old), the embryos have normally hatched and are easily observable. The larvae were rinsed twice in E3 before observation. Untreated control batches received only the solvent used for the drug stock solution. For teratogenicity assessment of caffeine, theophyllin and DCA, the surviving larvae were observed for morphological defects and the number of larvae presenting at least one morphological defect was reported as percentage of the surviving larvae. For LC50 and EC50 calculation, GraphPad Prism software was used.

### Manual image acquisition

Control or treated embryos were placed in a melt of E3 and methylcellulose, in a glass plate with 12 cavities (Hecht, Sondheim, FRG), one fish per well. Images were captured using an Olympus SZX10 stereo dissecting microscope coupled with an Olympus XC50 camera with a transmitted light illumination, the light passing up from the condenser and through the embryo. The Olympus XC50 camera allows us to acquire 2575 × 1932 pixel resolution images with a size of 14,2 Mo in TIFF format. We used the same parameters for all acquisition sessions (exposure time = 17ms, contrast = 1.05, maximum luminosity, white balance, magnification = 1.60x). First test runs revealed the ability of the classification algorithm to classify the images according to the acquisition session, therefore we defined a rigorous protocol in order to avoid variations in acquisition adjustments and parameters inducing artifacts that could bias the image analysis algorithms [9, 10]. Besides the primary aspects, such as the control of luminosity and focus, we also paid a particular attention to the position of the fish and to the nature of the plates (*e.g.*: glass) in order to avoid light refraction problems, causing shadowed parts on the images that can disturb the analysis. Finally, we decided to include images from five independent acquisition sessions into the learning set (see also below).

### Image pre-processing

**Figure 1:**
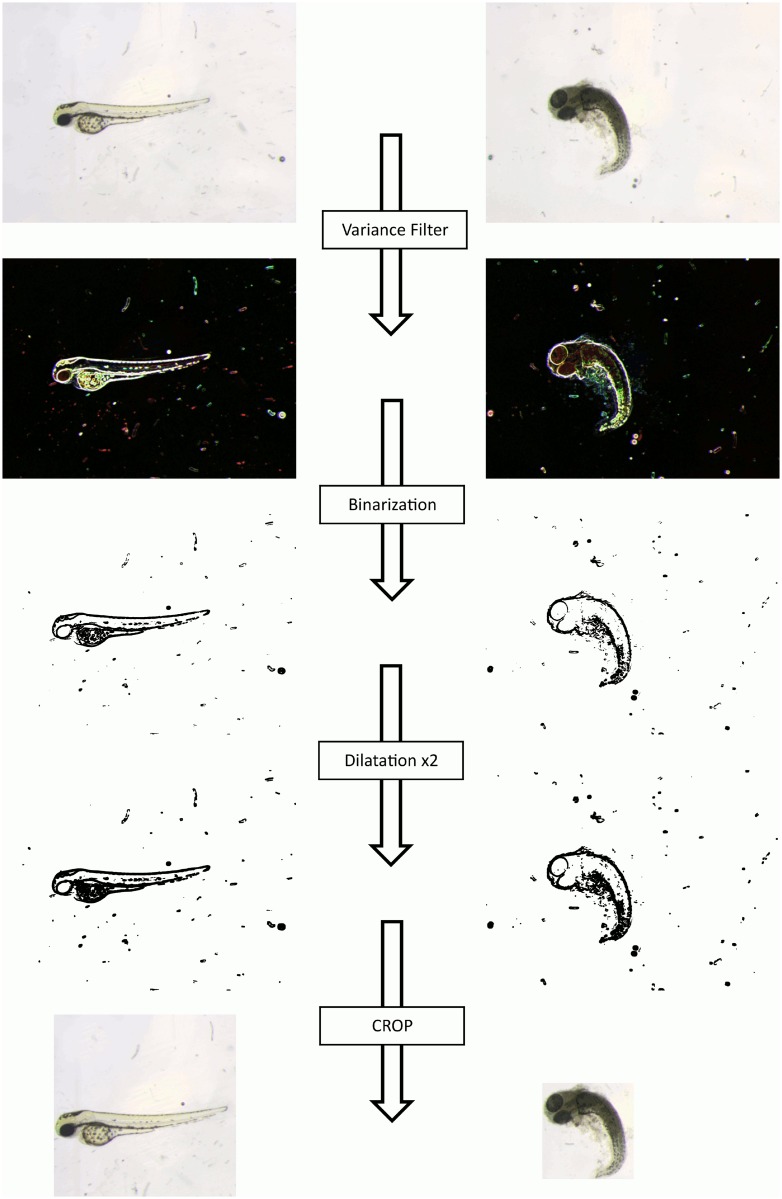
Image Preprocessing: From original image to the one used for classification. The original image is submitted to an ImageJ script (S1 Fig. for the code) which allows to automatically crop the zebrafish embryo into a square as far as possible. The embryo will be surrounded by a rectangle if it is placed near the sides of the original image. We use a connected-component labeling approach combined with different morphological transformations and binarizations, in order to localize the embryo.

To maximize the efficiency of our classification algorithm, larvae images were processed in order to be standardized before being classified. This pre-processing consists in removing parts of the image background that could contain noise unrelated to the phenotype to be detected and is performed using the ImageJ software (http://imagej.nih.gov/ij/). An algorithm has been developed within the ImageJ environment based on shape detection of the larvae (Fig. 1 and S1 Fig. for code). At this stage, images are first submitted to a series of morphological operations. First, a variance filter allowing to highlight edges in the images by replacing each pixel with the neighborhood variance is applied in order to highlight the edges of the objects within the image (larvae and/or debris), ignoring image borders. Images are then binarized to apply two dilatations in order to obtain a continuous outline surrounding each region. Finally, a connected-component labeling is used to obtain the object with the maximum area, to select and localize the larvae. If several connected components are found in the image, the largest one is assumed to be the larva, and its coordinates are kept. A supplementary test (ratio between height and width of the rectangle) is performed before cropping to check whether this region has a circular shape (as is the case for some phenotypes). Preliminary classification tests revealed that the best efficiencies were obtained if, at the end of this preprocessing, all larvae are displayed in the center of a square. In the case of a fish (region) with a circular shape, the region is cropped by the square directly surrounding it (Fig. 1, right). Otherwise, the region is placed into a square using the largest dimension of the surrounding rectangle (see algorithm in S1 Fig.). This algorithm fully works on all our images without damaging any of the embryos in term of shape or size.

### Image labeling—manual ground truths

Manual annotations of zebrafish phenotypes were performed using the CYTOMINE web-based application [11] (see also www.cytomine.be) that allows to upload microscopy images and annotate them according to user-defined vocabularies. In addition, each annotated defect is linked to a user-defined region of interest in the corresponding image.

### Supervised image classification

Supervised image classification methods exploit a dataset of labeled images to build a model (training phase) able to classify new, unseen images based on their visual content (testing phase).

### Training defect classification models by machine learning

We used a previously described machine learning based image classification algorithm [12] using dense random subwindows extraction in images, their description by raw pixel values and, finally the use of ensembles of extremely randomized trees [13] to classify these subwindows, and thence images by the joint exploitation of these subwindows’ classifications. Specifically, the method first extracts 1000 subwindows of random sizes and at random locations in each training image. Random sizes are controlled by two parameters: minimum and maximum sizes as a fraction of the total image size. These subwindows are resized to a fixed-size (in our case 32×32 pixels) and then described by raw pixel intensity values in a given color space. We used a normalized red-green-blue space (TRGB) where pixel value distributions were normalized within each subwindow for each RGB channel independently (by subtracting the mean and then dividing by the standard deviation). Then, an ensemble of extremely randomized trees is built with parameters T (the number of trees), N_min_ (the minimal size of the sample to split a node), K (the number of random tests evaluated at each test node) and the type of tests at each node, SIMPLETHRES (single pixel value thresholding) or DIFFNEIGHBOR (thresholding the difference of one pixel with one of its 8 neighbors). Once an ensemble of trees is built, it can be used in two modes: either to directly classify subwindows hence images (C mode), or to build image features (based on subwindow frequencies in terminal nodes) classified by a linear SVM method (BAGS mode) also trained on the training set.

### Prediction using the trained classifiers

To classify a test image, subwindows are as well extracted randomly according to the parameters used for training the classifier and propagated into the trees built at the learning step. In the direct classification mode (C mode), for each subwindow, and for each tree, class probability estimates are predicted and then aggregated by averaging both over the subwindows and the ensemble of trees; the most probable class of the resulting vector of probabilities is then assigned to the image. In the BAGS mode, an image descriptor is built based on the frequencies of appearance of its subwindows in each leaf of each tree of the ensemble. This high-dimensional descriptor is propagated into the linear SVM model, which outputs a final class decision for the image.

### Parameter tuning

The values of the main parameters (ranges of sizes of randomly extracted subwindows, type of tests at each internal node, classification mode) of the algorithm were optimized by cross-validation on the learning set. For each step of this internal cross-validation, we chose to use 2/3 of the learning set images to train a model and the remaining 1/3 to estimate its accuracy. To get more stable estimates, results were averaged over 5 to 75 runs with randomized 2/3–1/3 splits. Then, for each classifier (several classifiers are built to cover the different phenotypes; see below), we kept the parameter values that achieved the best cross-validated recognition rate for the corresponding prediction problem, and with these settings we then built a new model on the whole balanced learning sample, and applied it on the test images in order to assess their accuracy.

## Results

### Treatments

In order to obtain a collection of different morphological defects, zebrafish embryos were treated with increasing concentrations of several chemicals, known to cause specific defects on zebrafish larvae except thallium (Table 1). We chose to treat the embryos starting at 48 hpf (2 dpf) and to analyze the effects at 3 dpf, a period that we previously showed to be highly sensitive both concerning lethality and teratogenicity [14].

**Table 1:** Summary of the effects of the substances used to intoxicate the zebrafish embryos.

**Substances**	**Family**	**Observed Defects**
Acetaminophen	Cardioactive	Tail, Heart and Yolk Sac malformations
Propanolol	Cardioactive	Large pericardial edemas, weak pigmentation and tail curvatures
Amiodarone	Cardioactive	Failure of cardiac valve formation
Thallium	Heavy Metal	High toxicity but morphological effects on the zebrafish embryos are still unrecognized
Methylmercury (MeHg)	Heavy Metal	Tail fin fold defects and abolishes the tail fin primordium
Lead acetate (PbAc)	Heavy Metal	Induces malformations such as uninflated swim bladder, bent spine and yolk-sac edema
Zinc sulfate (ZnSO4)	Heavy Metal	Causes pathological alterations in isolated fish erythrocytes and induces abnormal embryogenesis, low hatchability, delayed hatching, and reduction of newly hatched larvae, and a poor survival ratio
Valproic acid (VPA)	Anticonvulsant and Mood-stabilizing	Causes a ventrally curved body axis and pericardial edema

A first class of compounds belongs to the cardioactive family. Acetaminophen is known to cause tail, heart and yolk sac malformations [15, 16], while propanolol exposure leads to large pericardial edema, weakened pigmentation and tail curvatures [17]. Finally, amiodarone is known to cause failure of cardiac valve formation in zebrafish embryos [18].

The second class belongs to the heavy metal family. Thallium is known for its high toxicity as a pollutant [19], even if its morphological effects on the zebrafish embryo are still unknown. Methylmercury (MeHg) causes tail fin fold defects and abolishes the tail fin primordium [20], while lead acetate (PbAc) can induce malformations such as uninflated swim bladder, bent spine and yolk-sac edema [21]. Zinc sulfate (ZnSO4) was shown to cause pathological alterations in isolated fish erythrocytes [22, 23] and abnormal embryogenesis, low hatchability, delayed hatching, a reduction of newly hatched larvae and a poor survival rate [24]. Finally, valproic acid (VPA) was shown to cause a ventrally curved body axis and pericardial edema [25, 26].

At the end of the treatment (3 dpf), the compounds were washed away and images were obtained of each individual larva in a lateral view. In order to test for robustness of the classification, eight completely independent acquisition sessions were organized on different days.

### Building the dataset

**Figure 2:**
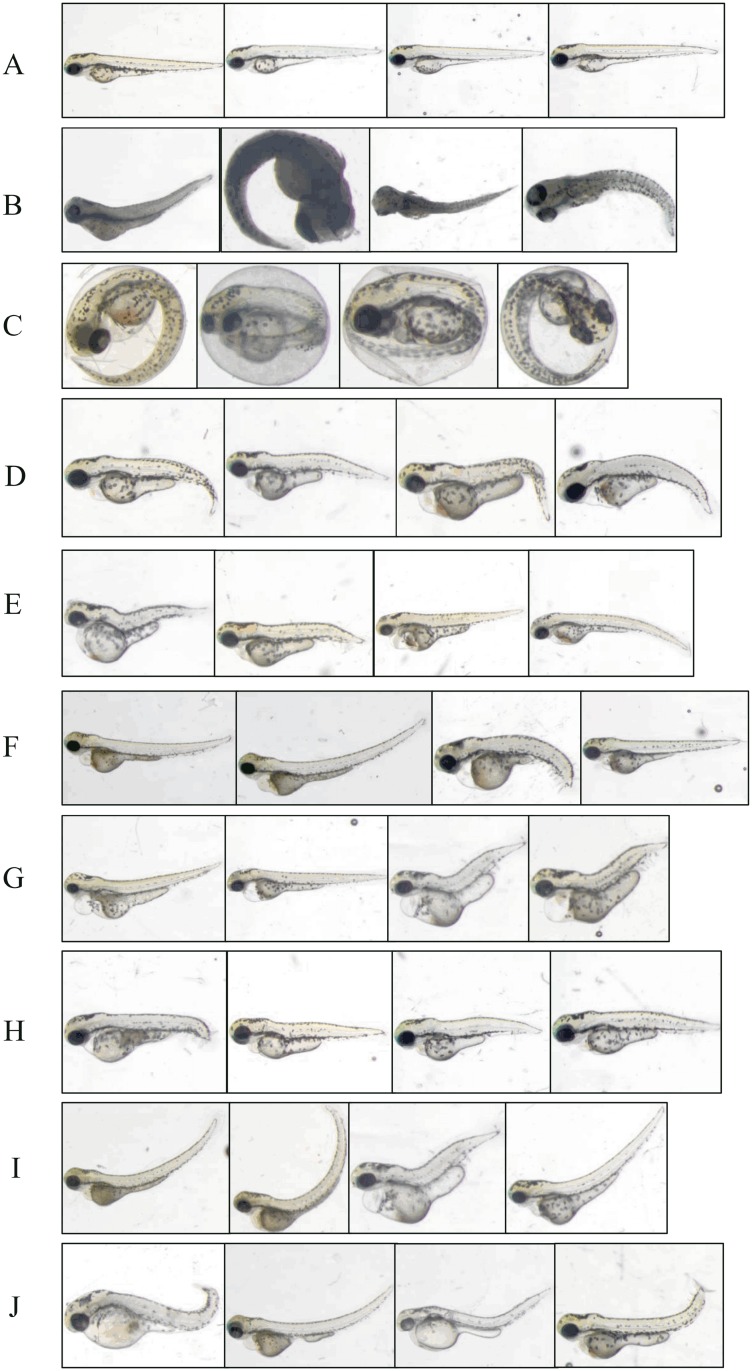
Examples of images representing all the analyzed phenotypes. “Without phenotype” defect, also called “Normal” embryos (A) are without any phenotype. The “Dead” phenotype (B) is shown as totally necrosed, while the “Chorion” phenotype (C) represents embryos that are still located in their chorion. In “Down Curved Tail” (D), the tail is obviously oriented downward compared to the horizontal. “Hemostasis” (E) presents a small amount of blood which can be located everywhere in the embryo (mainly in the head or in the pericardial area). “Necrosed Yolk Sac” (F) corresponds to a darker yolk compared to normal. In the “Edema” phenotype (G), an edema generally surrounds the anteroventral part of the fish. The “Short Tail” phenotype (H) describes a tail shorter than normal. “Up Curved Fish” (I) and “Up Curved Tail” (J) are two slightly different phenotypes. There is a curvature on the back of the embryo for “Up Curved Fish” (like a kind of lordosis), whereas the curvature is located on the tail for the “Up Curved Tail” phenotype.

In a first annotation round, every larva image (all phenotypes mixed randomly) was labeled by three biologists working independently who chose to assign one or more phenotypes according to their observation for each image. This first round served to define a vocabulary describing eleven defects that were observed in the image collection: “Normal”, “Dead”, “Chorion” probably indicating a general developmental delay, “Down Curved Tail”, “Hemostasis”, “Necrosed Yolk Sac”, “Edema”, and “Short Tail”. Finally, we defined “Up Curved Tail” and “Up Curved Fish”, depending on the location of the curvature, and «Up Curved Tail/Fish” for larvae presenting one of these two defects. Then, for each image and for each phenotype, the ground-truth was calculated by majority voting, *e.g.* a zebrafish was assigned the phenotype “Hemostasis” if at least 2 experts assigned that term to the embryo. Overall, 894 images have been manually acquired and analyzed one by one by the three experts who assigned roughly 7000 independent annotations. This first annotation round revealed the expert’s subjectivity, mostly due to the lack of rigorous phenotype definitions, most notably for the sensitive ones (*e.g.* edema and hemostasis) (overall agreement among the three experts 85–97% compared to the majority vote). We therefore organized consensus voting sessions: the three experts were asked to review their previous annotations all together in order to reach agreement on the phenotype(s) associated to each image. These sessions were repeated in order to have high-confidence learning sets. Finally, 870 images received a high consensus for the respective defect. Among these 870 images, 529, corresponding to five independent acquisition days, were integrated into the learning set. The remaining 341 images (3 additional acquisition days) were integrated into the test set. 24 images did not receive a consensus for at least one of the respective defects, and hence were removed from the dataset; they will be discussed later. Examples of all 11 defects are shown on Fig. 2 and a summary of the total number of images for each defect is given in Table 2 for both learning and test sets.

**Table 2:** Number of images by class (+) and (-) for each phenotype, in the learning set (LS) and the test set (TS).

**Learning Set (LS) and Test Set (TS): Summary of the Nb of images for each class**				
	**LS**		**TS**	
**Phenotypes**	Nb of (+) images	Nb of (-) images	Nb of (+) images	Nb of (-) images
**Dead**	114	415	53	288
**Chorion**	18	511	5	336
**Down Curved Tail**	11	518	16	325
**Hemostasis**	57	472	83	258
**Necrosed Yolk Sac**	167	362	11	330
**Edema**	160	369	54	287
**Short Tail**	49	480	149	192
**Up Curved Tail**	32	497	17	324
**Up Curved Fish**	64	465	13	328
**Up Curved Tail/Fish**	96	433	29	312
**Normal**	160	369	82	259

### Binary classification

In a first attempt to obtain automatic classification, we decided to build models for binary classification of each defect separately. The learning set for each specific defect was composed of those images presenting the defect (YES) against all the other images (NO).

A collection of binary models was built by varying the different parameters (see Material and Methods) and the optimal parameter combination was determined for each defect by cross-validation on the respective learning set. Systematically, T = 10 trees have been built for each binary test with K = 28 candidate splits performed per node, for all phenotypes. K = 28 was chosen as it is the default value suggested for color images [13] (*M* = 768, with *M* the total number of attributes describing subwindows). We built 10 trees since it is the minimal recommended value as previously assessed [12] (other tests have been performed with K = 128 and T = 100 but the results were not significantly improved). N_min_ was set to its recommended values: 1 for the classification mode “C” and 1000 for the classification mode “BAGS”. Table 3 shows the best parameter values (the classification mode [“C” or “BAGS”], the size of the random subwindows and the test type at each node [“SIMPLETHRES” or “DIFFNEIGHBOR”]) for each binary model as well as the achieved recognition rates on the learning set and the corresponding results on the corresponding independent test set using optimal binary models. All combinations of all the values for these parameters have been tested in the cross-validation step to determine the combination giving the best recognition rate for a given phenotype. The results (Table 3) reveal that one developmental anomaly, “Dead”, is identified with a near certainty (99.06%), while others, such as “Necrosed Yolk Sac”, “Up Curved Fish”, “Normal” and “Chorion” are still well classified (between 90.00%-95.15%). Phenotypes “Up Curved Fish/Tail”, “Short Tail”, “Down Curved Tail” and “Up Curved Tail” are a bit less well classified (between 80.43% and 89.94%), while “Hemostasis”, and “Edema” are not well detected at all by the classification algorithms (resp. 54.57%-73.85%).

**Table 3:** Summary of the classification results in cross-validation on the learning sets and on the independent test sets using the “All binary” or “Two-tier” method.

**Phenotype**	**Mode**	**Subw size (%)**	**Test Type**	**CV Rate on LS with “All binary”**	**Rate on TS with “All binary”**	**Rate on TS with “Two-tier”**
**Chorion**	BAGS	25–75	SIMPLETHRES	99.63% Chorion: 100% “-”: 99.26%	90.00% Chorion: 80% “-”: 100%	*Chorion: 100.00% Dead: 98.11% Others: 99.65%*
**Dead**	BAGS	10–75	SIMPLETHRES	99.73% Dead: 99.74% “-”: 99.74%	99.06% Dead: 98.11% “-”: 100%	*Dead: 98.11% Chorion: 100% Others: 99.65%*
**Down Curved Tail**	BAGS	50–90	DIFFNEIGHBOR	85.13% Down: 95.38% “-”: 74.87%	82.68% Down: 68.75% “-”: 96.61%	85.80% Down: 75.0% “-”: 96.61%
**Necrosed Yolk Sac**	BAGS	0–100	SIMPLETHRES	92.36% Necr.: 99.63% “-”: 85.09%	95.15% Necr.: 100% “-”: 90.30%	90.15% Necr.: 90.91% “-”: 89.39%
**Edema**	BAGS	10–90	DIFFNEIGHBOR	92.07% Edem.: 95.09% “-”: 89.06%	73.85% Edem.: 75.92% “-”: 71.78%	75.24% Edem.: 75.92% “-”: 74.56%
**Short Tail**	BAGS	25–90	DIFFNEIGHBOR	91.25% Short: 94.16% “-”: 88.33%	89.94% Short: 89.26% “-”: 90.62%	89.12% Short: 86.58% “-”: 91.67%
**Up Curved Fish**	C	25–75	SIMPLETHRES	96.19% UpFish: 99.04% “-”: 93.33%	92.04% UpFish: 92.31% “-”: 91.77%	95.42% UpFish: 100% “-”: 90.85%
**Up Curved Tail**	BAGS	25–90	DIFFNEIGHBOR	87.0% UpTail: 94.0% “-”: 80.0%	86.54% UpTail: 76.47% “-”: 96.60%	85.45% UpTail: 76.47% “-”: 94.44%
**Up Curved Tail/Fish**	C	0–90	SIMPLETHRES	94.84% UpFishTail: 98.84% “-”: 91.56%	80.43% UpFishTail: 72.41% “-”: 88.46%	78.55% UpFishTail: 68.96% “-”: 88.14%
**Hemostasis**	BAGS	25–90	DIFFNEIGHBOR	79.82% Hemo: 86.67% “-”: 72.98%	54.57% Hemo: 28.91% “-”: 80.23%	51.31% Hemo: 8.43% “-”: 94.19%
**Normal**	BAGS	10–75	SIMPLETHRES	97.54% Norm.: 98.87% “-”: 96.23%	91.09% Norm.: 98.78% “-”: 83.40%	91.09% Norm.: 98.78% “-”: 83.40%

For each phenotype (column 1), the optimal mode of classification (“BAGS” or “C”) (second column), the size of the extracted random subwindows (expressed in percentage of the size of the original image) (third column) and the kind of test type used for each node (SIMPLETHRES or DIFFNEIGHBOR)(fourth column) are given. “CV rate on LS” gives the results for each phenotype obtained in cross-validation on the corresponding learning sets (LS) with these parameters in the following form: global recognition rate, recognition rate of the specific phenotype, recognition rate of the corresponding “negative” phenotype. “Rate on TS” gives the results obtained with the same parameters on the corresponding independent test sets, respectively with the “All binary” or with the two-tier approach (three-class model followed by binary classification). Recognition rates are in % of the corresponding set.

### Two-tier classification

According to our first results, the two defects “Chorion” and “Dead” were identified with a near certainty. These two specific phenotypes, unlike other defects, are exclusive classes: by definition, fish belonging to these classes have no other phenotypes. We therefore decided to test a two-tier approach, where we would first sort-out those larvae presenting either the “Dead” or the “Chorion” phenotype, followed by a detection of the other defects if neither of these two phenotypes is detected by the classifier.

**Figure 3:**
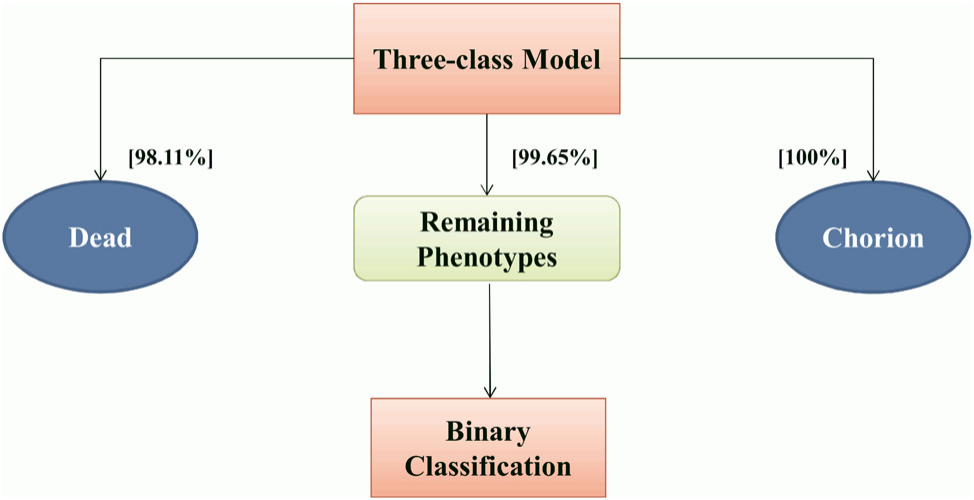
Two-tier pipeline. Schematic overview of the Two-tier approach for classification, also showing the recognition rates observed on the test set for the three-class model.

A three-class classification model was hence first built using the entire learning set to obtain three mutually exclusive classes: “Chorion”, “Dead” and “Others”. Note that the “Others” class includes both non-affected (i.e. “Normal”) larvae and those presenting at least one of the other defects. This first-tier classification model results in 100%, 98.11% and 99.65% of correct recognition rate, respectively for these three classes (Fig. 3), with in particular a very marked increase in the recognition rate of the “Chorion” phenotype from 90% to 100%. This very high success rate allowed us to consider the subsequent classification of the remaining phenotypes after putting aside the “Chorion” and “Dead” images.

Thus, images previously classified as “Others” by this first-tier classification algorithm composed the new datasets for detection of the remaining defects. New learning and test sets were constructed to build and evaluate new models for each phenotype by removing all the manually annotated “Dead” and “Chorion” images for the learning set and all the predicted “Dead” and “Chorion” images for the test set (S1 Table). Each new binary classification model was built for each of the remaining individual defects with the same parameters than those found optimal when building the corresponding defect model with “Dead” and “Chorion” images included. These models were applied to the new test sets, previously passed through the three-class model (S2 Table).

To compare the results of the “all binary” models and the two-tier approach using the three-class model followed by the new binary models, we aggregated the results of the two tiers into confusion matrices in order to obtain the global, final recognition rates. Indeed, false positive images for “Dead” and “Chorion” in the three-class test were missing from the “Others” phenotype, *i.e.* from the new test set for binary classification and thus represent false negatives for all true defects that they may contain. Conversely, false negative images for “Dead” and “Chorion” might actually be identified as false positives in the binary tests. This problem is minimal (only two images) due to the high recognition rates of the three-class model, but has to be considered when assessing the overall efficiency of the method. Taking these corrections into account, confusion matrices were constructed manually for the entire test set and for each defect (Table 3).

The observed classification accuracy for each phenotype reveals that this procedure significantly increased the recognition rates for “Chorion” (90% to 100%), slightly increased the recognition rates for “Down Curved tail” (82.68% to 85.80%), and “Edema” (73.85% to 75.24%), whereas it decreased recognition rates for “Necrosed Yolk Sac” (95.15% to 90.15%) and slightly decreased the recognition rates for “Up Curved Tail/Fish” (80.43% to 78.55%) and “Hemostasis” (54.57% to 51.31%) while leaving the others unchanged. Thus, we achieve really good results for “Dead”, “Chorion” and “Up Curved Fish” (95% > RecogRate > 100%), “Necrosed Yolk Sac” and “Normal” (90% > RecogRate > 95%), and good results for “Down Curved Tail”, “Up Curved Tail” and “Short Tail” defects (85% > RecogRate > 90%). Thus, the results are satisfying on the whole except for three phenotypes which remain really problematic: “Up Curved Tail/Fish”, “Edema” and “Hemostasis”.

We mentioned previously that 24 images did not reach consensus among expert annotations and were therefore not included in the learning and the test sets used in the previous experiments. However, we decided to use these images as a further test of our method on a set of particularly difficult images to see whether the experts could agree “*a posteriori* “with the annotation proposed for them by our classifiers.

These 24 images were hence run through the two-tier annotation pipeline. Their automatic annotations were then confronted for each image with the expert opinions, without revealing their previous opinions. The three-class model classifies all 24 images in the “Others” class, no “Chorion” or “Dead” embryo was present in this set, in perfect agreement with the experts. Agreement was also reached for 12 (out of 24) images with “Necrosed Yolk Sac”, 0 for “Down Curved Tail”, 11 for “Short Tail”, 4 for “Up Curved Tail” defects, respectively and 2 for the “Normal” phenotype. (Among all these, only 4 defects were spotted by the experts as potential false negatives.) For “Up Curved Fish” and “Up curved Fish/Tail”, the experts agreed on, respectively 8/22 and 4/8 annotated positive ones. As expected, agreement was low for “edema” 11/22 and “hemostasis” (3/12). These results are summarized in Table 4.

**Table 4:** Automatic classification of “difficult” images and “*a posteriori*” expert agreement.

	**Classified as (+) by algorithm**	**Expert agree**	**False negative?**
**Dead**	0	0	0
**Chorion**	0	0	0
**Necrosed Yolk Sac**	12	12	2
**Down Curved Tail**	0	0	1
**Short Tail**	11	11	0
**Up Curved Tail**	4	4	1
**Normal**	2	2	0
**Up Curved Fish**	22	8	2
**Up Curved Fish/Tail**	8	4	8
**Edema**	22	11	0
**Hemostasis**	12	3	2

24 “non consensus” images were first classified using the “Two-tier” method and then evaluated by experts for possible agreement. The second column shows the number of images that automatic classification identified as having the corresponding phenotype (+). These images were visualized by an expert and he provided an “*a posteriori*” opinion on the classification performed automatically. The number of images where he agreed with the automatic classification are shown on the third column. Finally, all images were again screened by the expert for all phenotypes to identify possible “false negative” images, their number is given in the last column.

### Model validation

Finally, in order to simulate a real-life toxicological experiment, new tests were performed to obtain several independent test sets, composed of new images collected manually using the same stereomicroscope and exactly the same parameters. The lethality and teratogenicity of the tested substances caffeine and theophylline have been previously characterized on zebrafish embryos after treatment between 48 and 72 hpf [14], while 3,4-dichloroaniline (DCA) is recommended as a toxicity standard in the OECD guidelines for the zebrafish embryotoxicity test [27]. Description of these compounds is given in in Table 5.

**Table 5:** Chemicals used to intoxicate embryos to build the validation set.

**Chemical**	**Stock Solution**	**Company**	**Molecular weight**
**Caffeine**	82,4mM	Sigma Aldrich	194.19
**Theophylline**	40,85mM	Sigma Aldrich	180
**3,4-Dichloroaniline (DCA)**	200mg/l	Sigma Aldrich	162.02

Name, concentration of stock solution, provider, chemical formula, and molecular weight are given.

Zebrafish embryos were intoxicated following the same protocol as previously described. We used theophylline (0.5mM, 1mM, 2mM, 5mM, 6mM), caffeine (0.5mM, 1mM, 3mM, 5mM, 7mM) and 3,4-dichloroaniline (4mg/l, 4.5mg/l, 5mg/l, 6mg/l, 7mg/l, 30mg/l, 40mg/l, 50mg/l, 60mg/l, 70mg/l) with an untreated control batch in each case. Then, new images were acquired manually, following the same protocol as previously (see *Manual Image Acquisition* section). Overall, 1191 images have been acquired and submitted to our two-tier classification pipeline without any retraining. For clarity, this new test set will be called “Validation Set”. Ground truth was established by manual observation of images.

### Three-class model

The validation set images were passed through the 3 class model to sort into “Chorion”, “Dead” and “Others” phenotypes. The results (Table 6) reveal an overall accuracy of 93.39% with a slight (about 10%) confusion between “Chorion” and “Dead” phenotypes. Most importantly, no image classified by experts as “Chorion” or “Dead” are injected into the “Others” phenotype, thus showing the reliability of our system concerning the exclusion of these two exclusive phenotypes.

**Table 6:** Summary of the classification results on validation set after being classified by the Two-tier approach.

	**Predicted « Chorion »**	**Predicted « Dead »**	**Predicted « Others »**	**Recognition Rate**
**True « Chorion »**	108	11	0	108/119 = 90,8%
**True « Dead »**	18	197	0	197/215 = 91,6%
**True « Others »**	1	18	838	838/857 = 97,8%

The number of predicted phenotypes and overall prediction rates are given for each of the three “true” subsets.

### Binary classification models

To go on simulating a real life toxicological experiment, only images classified by the three-class model as “Others” were injected into the various, previously constructed binary models. The proportions of each observed defect at the different compound concentrations are shown in S3–S5 Tables, both by manual (M) or automatic (A) observation. Comparison between manual and automatic classification reveals in general a good agreement with the exception of “Edema” and “Hemostasis”, as already expected. One remarkable exception is the abnormally high proportion (28/99) of “Necrosed Yolk Sac” observed in the untreated control for DCA (S5 Table), which seems to remain constant at all concentrations.

**Figure 4:**
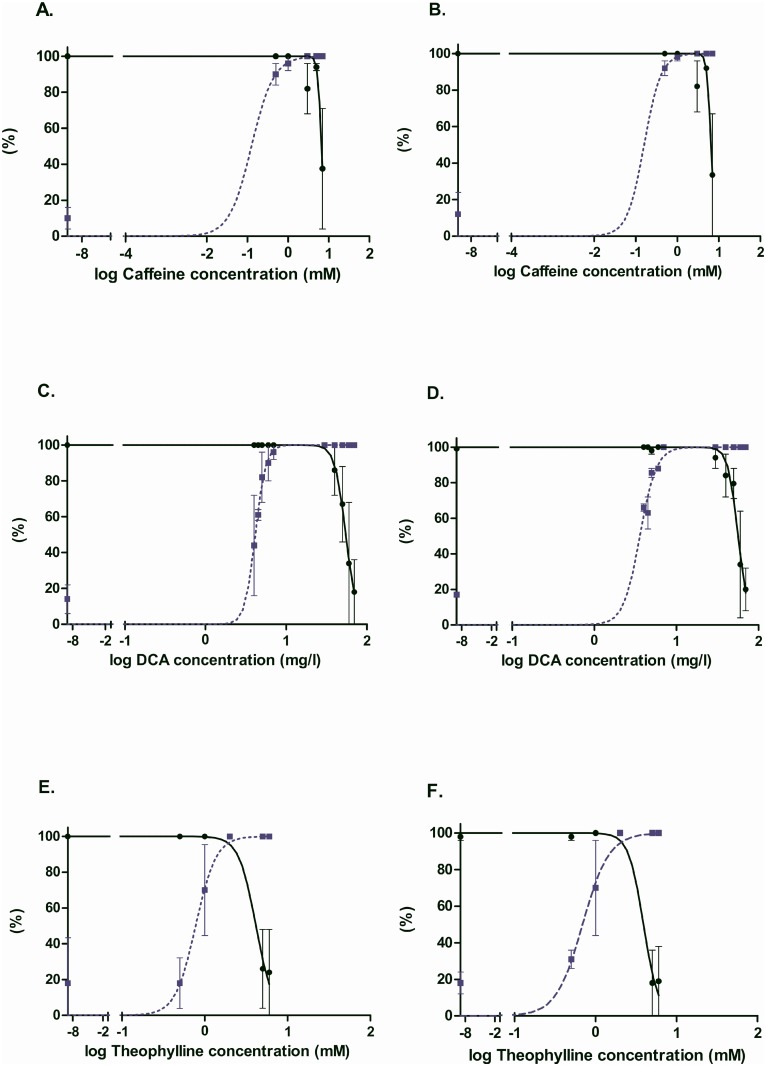
Dose-response curves for caffeine, theophylline and DCA. Survival and morphological defects of larvae intoxicated from 2dpf to 3dpf. The fraction of surviving larvae is represented by the “Survival” curve on each graph by a LC50 curve. The EC50 (teratogenicity) curve is drawn according to the results given by the “Normal” phenotype, as the fraction of surviving larvae. A, C, E graphs have been obtained on the basis of manual observations, whereas B, D and F graphs are based on automatic analysis.

Toxicological studies generally focus on the dose-response curves for survival and teratogenicity in order to deduce, respectively LC50 and EC50 values as well as the teratogenicity index TI (=LC50/EC50). Teratogenicity in this case means the presence of any defect in the surviving embryos. Comparing the manual and automatic dose response curves (Fig. 4), we observe a remarkable agreement of the obtained graphics. Furthermore, comparison of the deduced LC50, EC50 and TI values for the manual and automatic analyses and, for caffeine and theophylline with those previously published under the same conditions, reveals an excellent agreement, clearly within the range of experimental error (Table 7). Actually, conclusions about the effects of these compounds on zebrafish embryo development could have been directly deduced from the curves built from the automatic observations.

**Table 7:** Comparison of the deduced LC50, EC50 and TI for caffeine, DCA and theophylline.

	**log(LC50)**		**log(EC50)**		**log(TI)**		**log(TI) Litt**
	**Manual**	**Auto**	**Manual**	**Auto**	**Manual**	**Auto**	
**Caffeine**	0.82 ± 0.03	0.81 ± 0.02	-0.9 ± 0.7	-0.8 ± 1.0	1.72± 0.73	1.61± 1.0	1.26±0.05
**DCA**	1.74 ± 0.02	1.75 ± 0.02	0.62 ± 0.02	0.56 ± 0.03	1.12± 0.04	1.19± 0.05	Not available
**Theophylline**	0.62 ± 0.07	0.59 ± 0.07	-0.11 ± 0.05	-0.16 ± 0.01	0.73± 0.12	0.75± 0.1	1.06±0.04

This table presents all the log(LC50) and log(EC50) values processed on the manual observations and on the values processed by our classification pipeline. These values are compared for each chemical and finally log(TI) (TI = teratogenic index) are calculated this way: log(TI) = log(LC50)-log(EC50). According to the bibliography and also to the results given by our automatic classification, we can conclude that caffeine theophylline and DCA clearly revealed their teratogenicity (log(TI) > 0) on the basis of the results achieved by the automatic analysis.

## Discussion

Various recent works tackled the problem of automated analysis of zebrafish phenotypes following toxicological treatments [28–31]. One study determined the mortality rate due to various toxicant concentrations using images of treated embryos by extracting image features (*e.g.* variance of pixel values) to distinguish two very obvious classes, dead or alive embryos. Classification using the Matlab Gait-CAD toolbox was shown to be in good correlation with that from experts [28], however more complex defects were not addressed. Another work describes a way to automatically obtain images of zebrafish embryos using a motorized microscope, but leaves classification into phenotypes manual [29]. Other authors [30] developed an automatic system for data acquisition and embryo analysis in multi-well plates, however extensive manual intervention is still needed on a case-by-case basis to produce analysis routines using several image segmentations. More recently, an approach was proposed based on automatically acquired images, using a high-throughput microscope, followed by automatic classification into three basic phenotypes (hatched, unhatched and dead) using an algorithm based on MPEG-7 image descriptors and Support Vector Machines [32]. Our group proposed an automatic method to localize points of interest in zebrafish images that were taken manually [33]. Finally, a multi-thread system was presented that can simultaneously process multiple zebrafish larvae placed in a capillary, coupled to image recognition algorithms that fully automate manipulation of the animals, including their orientation and positioning regions of interest within the microscope’s field of view, however no solution to detect specific defects of the larvae is proposed [34, 35]. Recently, a method was described to extract body curvature along the length of an adult, swimming zebrafish from video data from a dorsal view {Cheng, 2014 #4248}. Another study describes automatic data acquisition of transgenic fluorescent larvae coupled to extraction of the body length as a single end-point for rapid assessment of a chemical’s toxicity {Lantz-McPeak, 2014 #4253}. Taken together, these studies essentially illustrate the difficulty to consider more specific and complex phenotypes. It clearly appears that the aim of a fully automatic procedure to capture images of zebrafish larvae and classify/quantify their phenotypes is not yet reached [6]. Existing solutions for image capture have to be adapted for high throughput applications, usability and cost-effectiveness. Defect recognition capabilities remain restricted, either by the narrow number of basic phenotypes considered, by the manual intervention still required for image classification or by a weak validation performed on a very small number of samples and/or image acquisition sessions.

Here, we propose a robust and high-performance automatic computer-based methodology to detect specific defects in images of zebrafish populations. Our approach succeeds in automated classification of embryo populations thanks to optimizations of algorithms based on machine learning and image processing. We show that the use of supervised learning algorithms for the classification of various defects is sensible.

Our final pipeline consists of a two-tier approach starting with a three-class classification model first sorting out images of “Dead” or “Chorion” embryos, before detecting other defects. These two phenotypes are highly relevant for toxicological studies, they are exclusive of other defects, and their identification with our methods is highly reliable. Moreover, the “Chorion” phenotype at this stage represents a clear delay in the developmental process, while more severe developmental timing defects (*e.g.* in epiboly, segmentation, …) might actually result in a “Dead” larvae. This step is similar to the one proposed previously [32] for classifying hatched, unhatched and dead embryos. When we evaluated our algorithms using these publicly available images (as JPEG files), following the protocols described above, we obtained slightly better results than their model: internal cross-validation on their training set (1.12%±1.09 vs 2.6%±0.95 error rate), and evaluation on their independent test set (3.12% vs 6.25% error rate, average processing time per image with single-threaded code: 0.3927s). When we tested our three-class model on our own validation set, obtained in completely independent experiments, we observed an occasional confusion between “Dead” and “Chorion”. Observation of the misclassified images shows that the confusion between “Chorion” and “Dead” phenotypes can be attributed to cases where the embryo is already half necrosed while still in the chorion (S2 Fig.). Manual observation may reveal some movement or heart beat of the embryo, leading to the classification alive and “Chorion”, but the partial necrosis in the still image misleads the classification algorithm into “Dead”. Inclusion of video/time-lapse into the classification process could help to avoid such mistakes, however we feel that such an approach would introduce complication with little benefit. Conversely, some dead embryos remain in a highly curled posture that may be assimilated by the classification algorithm as an unhatched, “Chorion” embryo. This confusion did however not significantly affect the survival and teratogenicity dose-response curves, as these rare mistakes compensated for each other. Most importantly, this confusion had no impact on the “Other” phenotype, thus allowing proper progression of our pipeline with the other defect identifications.

The binary models performed in general really well. Two defects, “Up Curved Tail” and “Necrosed Yolk Sac” ranged between 85–88% agreement with experts. Inspection of the classified images indicated that this relatively low agreement rate between automatic and expert classification for these two sensitive phenotypes might actually reflect the subjectivity of expert classification (S3 Fig.), rather than a failure of automatic classification. Thus, automatic classification might be useful to obtain a more objective evaluation as compared to human analysis. This could be especially true for low concentrations of toxicants, where the observed defects might be less severe and thus more dependent on subjective evaluation. Finally, two defects caused serious problems to the learning algorithms, “Edema” and Hemostasis”. This lack of recognition probably results from the more localized nature of these defects, as compared to the other, more general phenotypes. We are presently pursuing an approach using the localization information given by the expert annotation in the CYTOMINE environment to further refine our classification algorithms, either by focusing on the precise location of the defect or by focusing the learning phase on sub-images of the defect.

In the binary classification models, we included “Normal” as a specific phenotype, which was recognized at rates ranging between 90–95% and was used for drawing the dose-response curves in the validation experiment. Another logical way to evaluate the presence of this quite specific phenotype would be to consider that each image that has never been classified in the positive class for any phenotype belongs to the “Normal” class. However, in the tests that we performed using this method, it appeared that this procedure tends to accumulate errors made by the other classifiers and thus could result in extensive over- or under-estimation of the proportion of “Normal” phenotypes (data not shown). Especially the bad recognition of “Edema” and “Hemostasis” could interfere by delivering high numbers of false positives or negatives. Direct recognition of the “Normal” phenotype seems to be more robust against this bias. Note that, following OECD guidelines, under-estimation of the “Normal” phenotype in control, untreated populations might result in purely rejecting the experiment and thus time-consuming, costly and unnecessary repetition of the experiment. The binary model for recognition of the “Normal” phenotype seems to be the best solution in our setting.

In conclusion, our two-tier automatic classification pipeline already gives promising results in the analysis of 9 different defects out of 11 tested, allowing to anticipate that other morphological abnormalities could also be classified. In the future, we will focus on extending our classification method to other, more subtle or more localized defects.

One important question arising at this point is whether our pipeline is portable to other laboratories. We could show, using the validation set, that the method is very robust within a single laboratory, using the same equipment, settings and personal to obtain images at very different time points. This robustness is probably due to the fact that we used images obtained on 5 different acquisition days for the learning sets, thereby eliminating bias introduced by unrecognized differences in acquisition conditions. Direct use of the established models would probably require the use of the same, or similar instrumentation and settings in another laboratory. Alternatively, the CYTOMINE web-based application [11] can be used to gather specific new learning sets of appropriate quality, hence specific models could be constructed using the procedures described here. We are currently developing a user-friendly interface to make this functionality available to outside users. Another interesting application of such an objective classifier, trained over a representative set of labs or over a certain time span in a single lab, could be to monitor/arbitrate the variability in practice and performance between laboratories or in one laboratory over time.

The high success rate of our classification pipeline opens the way to automatic recognition of other features. Machine learning does not require any preconception concerning the feature to be recognized, thus integration of novel phenotypes will only be limited by the ability of experts to build appropriate learning sets. In high-content screening applications on cultured cells, fluorescent images are extensively used and automatic analysis has been discussed [36]. The transparency of zebrafish larvae is also used for fluorescent applications, thus we could possibly extend our analysis method to this kind of images. Furthermore, our method is not specific to zebrafish, we could envisage a general approach which could be extended to other organisms (e.g.: *drosophila melanogaster*, *C. elegans*).

## Supporting Information

S1 Fig Source code of the automatic image preprocessing. This macro has been written in JAVA meta-language in the ImageJ environment.
(TIFF)

S2 Fig Examples of images annotated as “Chorion” and “Dead” phenotypes misclassified by the two-tier approach. This Fig. shows the confusion made by the two-tiered approach between “Chorion” and “Dead” classes for some sensitive images. In column “A”, three images are annotated as belonging to the “Dead” class and classified by the two-tiered approach as “Chorion” because of their rounded shape and in some cases, the not totally necrosed embryo. On the other hand, column “B” shows examples of images annotated as “Chorion” but classified as “Dead” because of the necrosed aspect of the embryos.
(TIFF)

S3 Fig Examples of sensitive images for “Necrosed Yolk Sac” and “Up Curved Tail” phenotypes. This Fig. shows examples of images where the impact of the subjectivity of the experts during image annotation could explain the weak classification results for the two phenotypes “Necrosed Yolk Sac” and “Up Curved Tail”. Columns “A” and “B” show examples of images annotated as belonging, respectively to the “Necrosed Yolk Sac” and the “no_Necrosed Yolk Sac” classes. These images are actually very similar even if they were annotated as belonging to opposite classes. The same observation is made for images in columns “C” and “D”. Images in “C” are annotated by experts as belonging to the “Up Curved Tail” class whereas images in “D” are annotated as not belonging to the “Up Curved Tail” class.
(TIFF)

S1 Table Number of images in the Learning (LS) and Test Sets (TS) for binary classification in the Two-tier approach. After removal of the “Dead” and “Chorion” phenotypes from the Learning Set and after removal of the images classified as “Chorion” or “Dead” by the three-class model from the Test Set, there are less images than previously (Table 2).
(DOCX)

S2 Table Results of binary model classification on the independent Test set for the two-tier approach. Binary classification models were built for each of the remaining individual defects with the same parameters used to build the models with “Dead” and “Chorion” and these optimized models were applied to the new test set, previously passed through the three-class model. The numbers are given without correcting for the impact of the classification rates of the “Chorion” and “Dead” phenotypes in the three-class model.
(DOCX)

S3 Table Summary of the proportions of each observed defect (resp. Caffeine—Theophylline—DCA) at the different compound concentrations, both by manual (M) or automatic (A) observation. (D) = “Dead”, (C) = Chorion, (DT) = “Down Curved Tail”, (H) = “Hemostasis”, (NY) = “Necrosed Yolk Sac”, (E) = “Edema”, (ST) = “Short Tail”, (UF) = “Up Curved Fish”, (UFT) = “Up Curved Fish/Tail”, (UT) = “Up Curved Tail”, (N) = “Normal”. Each proportion is given as the number of larvae affected by the corresponding phenotype relative to the number of surviving fish. This latter number is very low at close to lethal concentrations, therefore dose-response curves and statistical analysis cannot be deduced in these cases.
(DOCX)

S4 Table Summary of the proportions of each observed defect (resp. Caffeine—Theophylline—DCA) at the different compound concentrations, both by manual (M) or automatic (A) observation. (D) = “Dead”, (C) = Chorion, (DT) = “Down Curved Tail”, (H) = “Hemostasis”, (NY) = “Necrosed Yolk Sac”, (E) = “Edema”, (ST) = “Short Tail”, (UF) = “Up Curved Fish”, (UFT) = “Up Curved Fish/Tail”, (UT) = “Up Curved Tail”, (N) = “Normal”. Each proportion is given as the number of larvae affected by the corresponding phenotype relative to the number of surviving fish. This latter number is very low at close to lethal concentrations, therefore dose-response curves and statistical analysis cannot be deduced in these cases.
(DOCX)

S5 Table Summary of the proportions of each observed defect (resp. Caffeine—Theophylline—DCA) at the different compound concentrations, both by manual (M) or automatic (A) observation. (D) = “Dead”, (C) = Chorion, (DT) = “Down Curved Tail”, (H) = “Hemostasis”, (NY) = “Necrosed Yolk Sac”, (E) = “Edema”, (ST) = “Short Tail”, (UF) = “Up Curved Fish”, (UFT) = “Up Curved Fish/Tail”, (UT) = “Up Curved Tail”, (N) = “Normal”. Each proportion is given as the number of larvae affected by the corresponding phenotype relative to the number of surviving fish. This latter number is very low at close to lethal concentrations, therefore dose-response curves and statistical analysis cannot be deduced in these cases.
(DOCX)
