# Supplementary material for: Phenotype Classification of Zebrafish Embryos by Supervised Learning
Source: PLoS One. 2015 Jan 9;10(1):e0116989. doi: 10.1371/journal.pone.0116989 (PMC4289190; doi:10.1371/journal.pone.0116989)
Supplement: S1 Table — After removal of the “Dead” and “Chorion” phenotypes from the Learning Set and after removal of the images classified as “Chorion” or “Dead” by the three-class model from the Test Set, there are less images than previously (Table 2). (DOCX) [file pone.0116989.s004.docx]

**Table S1**

| **Nb of images for each class after removal of "Dead" and "Chorion": Learning (LS) and Test (TS) set.** | | | | |
| --- | --- | --- | --- | --- |
|  | LS | | TS | |
| **Phenotypes** | Nb of (+) images | Nb of (-) images | Nb of (+) images | Nb of (-) images |
| **Down Curved Tail** | 11 | 386 | 16 | 267 |
| **Hemostasis** | 57 | 340 | 83 | 200 |
| **Necrosed Yalk Sac** | 167 | 230 | 11 | 272 |
| **Pericardial Edema** | 160 | 237 | 54 | 229 |
| **Short Tail** | 49 | 348 | 149 | 134 |
| **Up Curved Tail** | 32 | 365 | 17 | 266 |
| **Up Curved Fish** | 64 | 333 | 13 | 270 |
| **Up Curved Tail/Fish** | 96 | 301 | 29 | 254 |
| **Normal** | 160 | 237 | 82 | 201 |
