# Supplementary material for: Phenotype Classification of Zebrafish Embryos by Supervised Learning
Source: PLoS One. 2015 Jan 9;10(1):e0116989. doi: 10.1371/journal.pone.0116989 (PMC4289190; doi:10.1371/journal.pone.0116989)
Supplement: S2 Table — Binary classification models were built for each of the remaining individual defects with the same parameters used to build the models with “Dead” and “Chorion” and these optimized models were applied to the new test set, previously passed through the three-class model. The numbers are given without correcting for the impact of the classification rates of the “Chorion” and “Dead” phenotypes in the three-class model. (DOCX) [file pone.0116989.s005.docx]

**Table S2**

| **Summary of the Results on Independent Test Set** | | | | |
| --- | --- | --- | --- | --- |
|  | **Parameters** | | |  |
| **Phenotype** | **Mode** | **Subw Sizes (%)** | **Test Type** | **Rate (%)** |
| Down Curved Tail | BAGS | 50-90 | DIFFNEIGHBOR | 82.12%  Down : 68.75%  Ok : 95.50% |
| Necrosed Yolk Sac | BAGS | 0-100 | SIMPLETHRES | 88.65%  Necr. : 90.91%  Ok : 86.40% |
| Pericardial Edema | BAGS | 10-90 | DIFFNEIGHBOR | 72.24%  Edem. : 75.92%  Ok : 68.56% |
| Short Tail | BAGS | 25-90 | DIFFNEIGHBOR | 88.66%  Short : 89.26%  Ok : 88.06% |
| Up Curved Fish | C | 25-75 | SIMPLETHRES | 94.44%  UpFish : 100%  Ok : 88.89% |
| Up Curved Tail | BAGS | 25-90 | DIFFNEIGHBOR | 85.04%  UpTail : 76.47%  Ok : 93.61% |
| Up Curved Tail/Fish | C | 0-90 | SIMPLETHRES | 77.59%  UpFishTail : 68.96%  Ok : 86.22% |
| Hemostasis | BAGS | 25-90 | DIFFNEIGHBOR | 51.22%  Hemo : 8.43%  Ok : 94% |
| Normal | BAGS | 10-75 | SIMPLETHRES | 88.94%  With. : 98.78%  Ok : 79.10% |
