# Supplementary material for: Phenotype Classification of Zebrafish Embryos by Supervised Learning
Source: PLoS One. 2015 Jan 9;10(1):e0116989. doi: 10.1371/journal.pone.0116989 (PMC4289190; doi:10.1371/journal.pone.0116989)
Supplement: S4 Table — (D) = “Dead”, (C) = Chorion, (DT) = “Down Curved Tail”, (H) = “Hemostasis”, (NY) = “Necrosed Yolk Sac”, (E) = “Edema”, (ST) = “Short Tail”, (UF) = “Up Curved Fish”, (UFT) = “Up Curved Fish/Tail”, (UT) = “Up Curved Tail”, (N) = “Normal”. Each proportion is given as the number of larvae affected by the corresponding phenotype relative to the number of surviving fish. This latter number is very low at close to lethal concentrations, therefore dose-response curves and statistical analysis cannot be deduced in these cases. (DOCX) [file pone.0116989.s007.docx]

|  | **M** | **A** | **M** | **A** | **M** | **A** | **M** | **A** | **M** | **A** | **M** | **A** |
| --- | --- | --- | --- | --- | --- | --- | --- | --- | --- | --- | --- | --- |
| **D** | 0/50 | 1/50 | 0/49 | 1/49 | 0/50 | 0/50 | 0/50 | 0/50 | 37/50 | 42/50 | 37/48 | 40/48 |
| **C** | 0/50 | 0/49 | 0/49 | 0/48 | 0/50 | 0/50 | 0/50 | 0/50 | 0/13 | 0/8 | 0/11 | 0/8 |
| **DT** | 0/50 | 0/49 | 0/49 | 0/48 | 7/50 | 2/50 | 23/50 | 16/50 | 9/13 | 1/8 | 11/11 | 4/8 |
| **H** | 0/50 | 0/49 | 3/49 | 0/48 | 5/50 | 0/50 | 15/50 | 1/50 | 2/13 | 0/8 | 4/11 | 0/8 |
| **NY** | 3/50 | 8/49 | 0/49 | 12/48 | 10/50 | 12/50 | 20/50 | 29/50 | 13/13 | 8/8 | 11/11 | 8/8 |
| **E** | 8/50 | 6/49 | 7/49 | 7/48 | 11/50 | 17/50 | 18/50 | 28/50 | 7/13 | 8/8 | 7/11 | 8/8 |
| **ST** | 0/50 | 1/49 | 0/49 | 1/48 | 16/50 | 19/50 | 45/50 | 44/50 | 13/13 | 8/8 | 11/11 | 8/8 |
| **UF** | 3/50 | 4/49 | 2/49 | 5/48 | 3/50 | 5/50 | 3/50 | 9/50 | 0/13 | 3/8 | 0/11 | 1/8 |
| **UFT** | 3/50 | 6/49 | 5/49 | 7/48 | 5/50 | 10/50 | 5/50 | 11/50 | 0/13 | 4/8 | 0/11 | 2/8 |
| **UT** | 1/50 | 2/49 | 3/49 | 3/48 | 4/50 | 3/50 | 2/50 | 3/50 | 0/13 | 3/8 | 0/11 | 2/8 |
| **N** | 42/50 | 40/49 | 41/49 | 33/48 | 17/50 | 15/50 | 0/50 | 0/50 | 0/13 | 0/8 | 0/11 | 0/8 |
|  | **0mM** | | **0,5mM** | | **1mM** | | **2mM** | | **5mM** | | **6mM** | |
